# Urbanization-induced habitat fragmentation erodes multiple components of temporal diversity in a Southern California native bee assemblage

**DOI:** 10.1371/journal.pone.0184136

**Published:** 2017-08-30

**Authors:** Keng-Lou James Hung, John S. Ascher, David A. Holway

**Affiliations:** 1 Division of Biological Sciences, University of California, San Diego, La Jolla, California, United States of America; 2 Department of Biological Sciences, National University of Singapore, Singapore, Singapore; University of Guelph, CANADA

## Abstract

Despite a large number of ecological studies that document diversity loss resulting from anthropogenic disturbance, surprisingly few consider how disturbance affects temporal patterns of diversity that result from seasonal turnover of species. Temporal dynamics can play an important role in the structure and function of biological assemblages. Here, we investigate the temporal diversity patterns of bee faunas in Southern California coastal sage scrub ecosystems that have been extensively fragmented by urbanization. Using a two-year dataset of 235 bee species (n = 12,036 specimens), we compared 1-ha plots in scrub fragments and scrub reserves with respect to three components of temporal diversity: overall plot-level diversity pooled over time (temporal gamma diversity), diversity at discrete points in time (temporal alpha diversity), and seasonal turnover in assemblage composition (temporal beta diversity). Compared to reserves, fragments harbored bee assemblages with lower species richness and assemblage evenness both when summed across temporal samples (i.e., lower temporal gamma diversity) and at single points in time (i.e., lower temporal alpha diversity). Bee assemblages in fragments also exhibited reduced seasonal turnover (i.e., lower temporal beta diversity). While fragments and reserves did not differ in overall bee abundance, bee abundance in fragments peaked later in the season compared to that in reserves. Our results argue for an increased awareness of temporal diversity patterns, as information about the distinct components of temporal diversity is essential both for characterizing the assemblage dynamics of seasonal organisms and for identifying potential impacts of anthropogenic disturbance on ecosystem function through its effects on assemblage dynamics.

## Introduction

The alteration of natural habitats by human activities is generally acknowledged to reduce the abundance and diversity of organisms (e.g., [1–5]). However, our understanding of the consequences of anthropogenic disturbance remains incomplete because most studies that address the effects of disturbance pool or average data over the course of the study season (e.g., those cited in [5]). While studies that examined inter-annual variation in assemblages have yielded important insights into long-term temporal patterns in biodiversity [6–8], few studies have considered seasonal (i.e., intra-annual) variation exhibited by most biological assemblages. Given that seasonal variation in the identity, diversity and abundance of organisms plays an important role in the structure and function of communities [9–13], the effects of disturbance may be greatly underestimated without explicit consideration of such seasonal dynamics.

To account for how seasonal dynamics influence the response of an assemblage to disturbance, one may separate the assemblage’s diversity into temporal gamma, alpha, and beta components in a manner similar to the partitioning of spatial diversity [14]. When diversity is examined in this temporal framework, temporal gamma diversity pertains to data pooled across individual temporal samples from a given locality [15]. As such, temporal gamma diversity is equivalent to “site-level diversity,” one of the most commonly reported measures of diversity in assemblage- and community-level studies. Temporal alpha diversity pertains to the finest temporal scale in which sampling is conducted [16], providing insight into diversity at discrete points in time and allowing for analyses of temporal trends within a study site. Lastly, temporal beta diversity measures the degree to which individual temporal samples at a study site differ from one another with respect to the composition of taxa present, providing insight into the temporal turnover of the taxa that make up an assemblage [17]. While some popular indices of beta diversity are mathematically derived from measures of alpha and gamma diversity (e.g., [14,16]), recent advancements in the field of statistics have enabled measures of beta diversity that are mathematically independent of measures of alpha and gamma diversity, such as multivariate dispersion [18].

**Fig 1:**
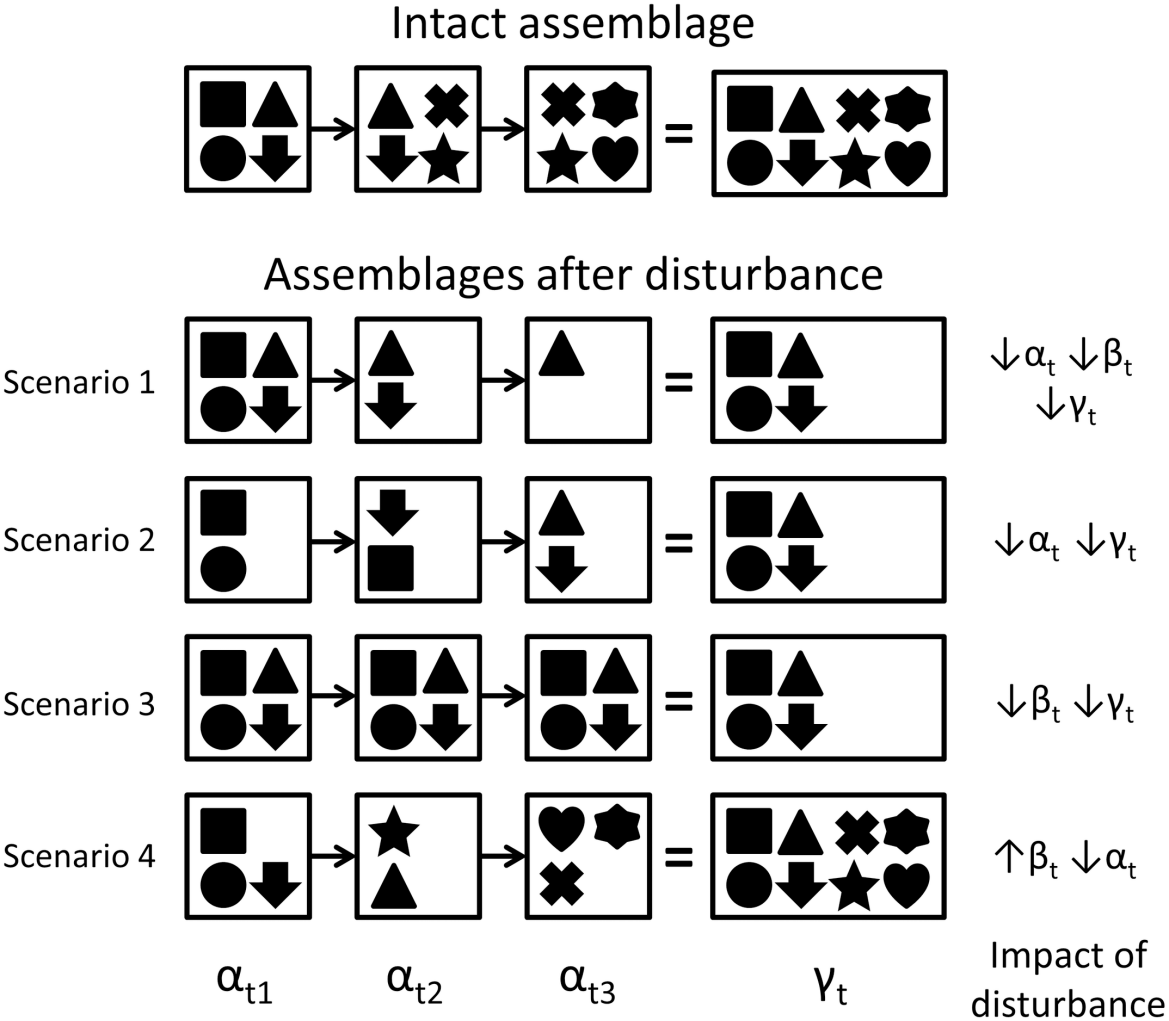
Hypothetical scenarios of disturbance impacting temporal gamma, alpha, and beta diversity of an assemblage. Each symbol represents a distinct taxon; α_t_ represents taxon richness at discrete time points (t1-3), β_t_ represents the turnover of taxa between time points, and γ_t_ represents taxon richness summed across time points.

Impacts of anthropogenic disturbance on temporal gamma diversity always result from changes in temporal alpha diversity, beta diversity, or both (Fig 1). Decreases in temporal alpha and beta diversity may be driven by different aspects of disturbance (e.g., [10]), and may have different implications for biological interactions and ecosystem function even if different patterns of temporal alpha and beta diversity loss lead to the same net change in temporal gamma diversity (Fig 1, scenarios 1–3). Trends in temporal alpha and beta diversity may also act in opposition such that temporal gamma diversity remains unchanged in spite of the profound alteration to temporal assemblage structure (Fig 1, scenario 4). Thus, isolating the mechanisms through which disturbance impacts an assemblage requires an examination of all three components of temporal diversity (e.g., [10]). Such approaches may also serve to identify the ecological effects that result from disturbance (e.g., [12,19]).

In this study, we investigated the impacts of urbanization-induced habitat fragmentation on the seasonal dynamics of a diverse native bee (Hymenoptera: Anthophila) assemblage over a two-year period. Bees represent an appropriate taxonomic group for studying how habitat fragmentation affects temporal dynamics because, like many other organisms that occupy seasonal environments, bees exhibit distinct periods of activity that differ among species with respect to both duration and timing of onset [20]. Previous research has demonstrated that anthropogenic disturbance may differentially impact bee species active in different seasons [21], and that temporal turnover in bee assemblages can contribute to among-habitat differences in site-level bee species richness [10]. Additionally, the key ecosystem function that bees perform (i.e., pollination) is influenced by the season-specific pollination effectiveness [22] and temporal complementarity [23,24] of individual bee species. An explicit consideration of temporal diversity patterns is thus necessary to assess how anthropogenic disturbance affects bee assemblage structure and to identify potential consequences for ecosystem function.

Here, we explicitly examined the seasonal dynamics of our focal bee assemblages by simultaneously evaluating their temporal gamma, alpha, and beta diversity. Our use of linear mixed-effects models and analyses of multivariate dispersion [18] distinguishes our study from previous work on temporal patterns in pollinator diversity, the majority of which has focused on quantifying the relative contributions of spatial versus temporal variation in structuring pollinator assemblages [10,25–28]. Our approach enabled us to address the following research questions: (1) does habitat fragmentation affect all three components of bee temporal diversity similarly, and (2) how do the effects of habitat fragmentation vary with time? Addressing these research questions allowed us to scrutinize the impacts of habitat fragmentation with a temporal resolution that would be unachievable by pooling temporal samples within study sites.

## Materials and methods

### Study system

Between April and August of 2011 and 2012, we documented bee assemblages in the coastal sage scrub ecosystems of San Diego County, California, USA, a global hotspot of bee biodiversity with over 500 bee species documented in the surrounding areas [29,30]. We established 1-ha study plots in coastal sage scrub habitat situated in (1) large natural reserves (internal area >> 500 ha), and (2) well-preserved habitat fragments (internal area < 120 ha, see S1 Table) embedded within the residential, urban matrix. In 2011, we surveyed four study plots in reserves and four study plots in fragments. In 2012, we surveyed seven study plots in reserves and 11 study plots in fragments. Details regarding the location and treatment classification (reserves or fragments) of each plot are provided in S1 Table. Many of our study plots are located in the same system of reserves and fragments included in earlier studies on the ecological effects of urbanization-induced habitat fragmentation [3,4,31], including bees sampled incidentally in pitfall traps [32]. Permission to conduct field research was obtained from the University of California, San Diego; the Otay-Sweetwater Unit and Tijuana River National Estuarine Research Reserve Unit of the US National Wildlife Refuge; the City of San Diego Open Space Parks Division and Real Estate Division; the City of La Mesa Open Space Division; and the City of Chula Vista Open Space Division.

### Data collection

One researcher (KLJH) employed bowl trapping and aerial netting [33] to sample bees at all study plots, on sunny days with light wind. Bowl traps consisted of plastic bowls 7 cm in diameter that were white (left unpainted) or painted fluorescent blue or fluorescent yellow and filled with ca. 60 ml of unscented detergent solution. Fluorescent paint was manufactured by Guerra Paint and Pigment Corp (“Fluorescent Blue” and “Fluorescent Yellow”) as fluorescent pigment dispersions and applied to plastic bowls using a water-based, flexible acrylic polymer emulsion. During each survey, 30 bowl traps were placed at a study plot before 0900 h and collected after 1500 h. Traps were placed on level ground in an alternating sequence of colors, deployed in two roughly linear transects originating from the corners of each plot and forming an “X” formation near the plot’s center. Traps were placed 5–10 m apart from one another and at least 1 m from the canopy of large shrubs to avoid being shaded. During aerial netting, one researcher walked throughout the study plot and examined blooming plants as well as presumed nesting substrates (bare ground and dead, woody plant material) for bees. Non-*Apis* bee species were collected regardless of whether they were on flowers, in flight, or in the vicinity of presumed nesting substrates. In 2011, surveys were performed ca. every 2–3 weeks at each study plot (n = 9 survey days per plot), during which time, 60-min bouts of netting were performed once between 0900 h and 1200 h and once between 1200 h and 1500 h (120 min total per plot per survey). In 2012, in order to accommodate a larger number of study plots, surveys were performed ca. every 3–5 weeks (n = 5 survey days per plot) and included only a single 60-min bout of netting at each plot during each survey. Although seven sites were sampled in both years (S1 Table), the level of sampling employed here seems unlikely to have altered bee assemblages during our study [34].

All collected bees were individually mounted and identified to species or morphospecies within genus using taxonomic keys and the reference collections of the American Museum of Natural History, UC Riverside Entomology Research Museum, California Academy of Sciences, UC Berkeley Essig Museum of Entomology, and UC Davis Bohart Museum of Entomology. Additionally, we also categorized each non-parasitic bee species as a pollen generalist or a pollen specialist based on whether it is documented to exclusively collect pollen from a single plant family. Data used to classify bees as generalists or specialists come from literature accounts for the species (or species group) [35] and its subgenus [20], as well as our own field observations.

Bee assemblages often reflect the richness, abundance, and temporal dynamics of their host plant assemblages [21,30]. Thus, concurrently with bee sampling, we documented the identities of insect-pollinated native plant species present in each plot in each year; in 2012 we also counted the number of blooming individuals of each plant species in each plot during each survey. We documented blooming plants by walking through pre-planned paths that allowed the observer’s field of view to cover the entirety of the study plot, as in [36], because many key plant species in our system are patchily distributed and because the thick growth of large, woody shrubs prohibited the use of random linear transects at many of our plots.

### Statistical analyses

We compared native bee assemblages in reserve versus fragment plots with respect to their temporal gamma, alpha, and beta diversity. We analyzed data from each year separately because of differences in sample size and sampling frequency. In order to avoid human biases associated with aerial netting (e.g., catch rate may be reduced at sites where the collector’s mobility is hindered by dense vegetation), our analyses include only bee specimens collected by bowl traps; however, inclusion of netted specimens in our analyses yielded qualitatively similar results. For analyses requiring species-level identification, we excluded 78 bee individuals (0.8% of individuals) not identifiable beyond genus. We also repeated all analyses at the genus level to ensure that particularly species-rich genera did not disproportionately influence our findings; the results of these additional analyses did not alter our main conclusions. Lastly, we verified that reserve and fragment plots did not differ with respect to the composition and temporal dynamics of insect-pollinated native plant assemblages, and that the plot-level compositions of bee assemblages were not spatially autocorrelated (see S1 Appendix).

All analyses were conducted in R version 3.3.1 [37]; packages *vegan* [38], *MASS* [39], *car* [40], and *nlme* [41] were used in visualizing and analyzing data. R scripts written for the main analyses are reported in S2 Appendix.

#### Temporal gamma diversity

We define temporal gamma diversity as the diversity of bees at a single study plot, pooled across all temporal samples (see [15]), with each sample representing the bee specimens collected at one study plot during a single day of data collection. We considered both species richness and assemblage evenness (Pielou’s *J*). In addition, we examined the proportion of bee individuals represented by generalist species (hereafter referred to as “generalist proportion”), as generalist bees can exhibit higher tolerance to anthropogenic disturbance compared to their specialist counterparts [42,43]. Lastly, we also examined the temporal gamma component of bee abundance. We used rarefaction (repeated for 1,000 iterations) in our analyses of species richness and assemblage evenness to account for among-plot variation in the number of bee individuals sampled. We used the lowest plot-level bee abundance recorded each year (n = 378 for 2011, n = 115 for 2012) as the number of individuals to subsample in our rarefactions. Bee abundance was calculated as the total number of bee individuals collected at each plot averaged across the number of temporal samples. Assemblage evenness and generalist proportion were logit-transformed prior to analysis as recommended by [44], and bee abundance was cube root-transformed to improve normality. We used Welch’s two-sample *t*-tests to compare fragment and reserve plots for all dependent variables listed above.

Given the dependence of bee diversity on the diversity and assemblage composition of their host plant assemblages [30], we also repeated each analysis with the temporal gamma richness of native insect-pollinated plants as an added independent variable (i.e., multiple regressions with treatment and plant richness as main effects). We then compared the Akaike Information Criterion (AIC) scores [45] of each pair of models with or without plant richness added. Compared to original models that did not include plant richness, models that included plant richness yielded qualitatively similar results in all cases but had poorer (i.e., more positive) or equivalent AIC scores; thus, we did not include plant richness in our final models.

#### Temporal alpha diversity

We define temporal alpha diversity as the diversity of bees collected in a single temporal sample at each study plot (see [10]). As in our analyses of temporal gamma diversity, we examined species richness, logit-transformed assemblage evenness, logit-transformed generalist proportion, and cube root-transformed bee abundance. In our analyses of species richness and assemblage evenness, we rarefied each temporal sample to 20 bee individuals (repeated for 1,000 iterations) to allow for unbiased comparisons between treatments and across temporal samples. In analyses requiring rarefaction, we excluded one sample from the 2011 dataset and nine samples from the 2012 dataset (including all samples from one fragment plot in which three of its five samples had fewer than 20 bees). We chose to rarefy to 20 individuals in order to minimize the number of data points to exclude while retaining sufficient resolution in our data.

To examine how bee assemblages in reserves and fragments differed over the course of the study period, we constructed linear mixed-effects models. This approach allowed us to quantify the direction of seasonal trends and to detect treatment-by-sample interactions, neither of which is possible for the additive diversity partitioning approach [16] used by most published studies that examined bee temporal alpha diversity (e.g., [10,26–28]). In each model, treatment (fragment vs. reserve), temporal sample (the Julian date on which sampling occurred), and their interaction were included as fixed effects, and study plot identity was included as a random effect to control for repeated sampling as in [21]. To account for possible non-linear relationships between dependent variables and Julian dates of temporal samples, we constructed second- and third-degree orthogonal polynomial models in addition to first-degree linear models for each dependent variable, and selected the model with the lowest AIC score. When alternative models yielded equivalent AIC scores (ΔAIC < 2), the model with the lowest degree was chosen. Lastly, as with our analyses of temporal gamma diversity, we repeated all analyses with the temporal alpha richness of native plants as an added independent variable (i.e., linear mixed-effects models including the main effects of treatment, temporal sample, and plant temporal alpha richness, and the interaction effect of treatment and temporal sample). Models that included plant richness yielded poorer AIC scores in all cases; thus, we did not include plant richness in our final models.

#### Temporal beta diversity

We define temporal beta diversity as the multivariate dispersion [17,18] of bee assemblages in distinct temporal samples from the same study plot. We chose this index because of its relative mathematical independence from measures of alpha and gamma diversity (i.e., it is not calculated from the difference or ratio between alpha and gamma diversity), as well as its capability to detect differences among assemblages in both species identity and relative abundance [18]. Accounting for abundance makes multivariate dispersion less sensitive to rare species, which often make up a large fraction of the total species richness in bee assemblages (e.g., [46,47]) but may contribute little to the pollination services rendered to plants [48]. For these reasons, multivariate dispersion is superior to the traditional approach of using multiplicative [14] or additive partitioning [16] for investigating bee temporal beta diversity (e.g., [10,26–28]) with respect to characterizing individual-level bee assemblage composition, as well as temporal turnovers in ecosystem function.

To calculate multivariate dispersion, we performed a non-metric multidimensional scaling (NMDS) ordination based on a dissimilarity matrix of abundance-weighted bee assemblages in all possible pairs of samples across all plots (dissimilarity was calculated using the Bray-Curtis index, see [18]). From this ordination, we calculated the multidimensional centroid of the samples from each plot, and then computed the mean distance between each plot’s centroid and its constituent samples. The resulting dispersion score for each plot thus measures the degree to which the species composition of each plot’s bee assemblage turns over through time. Dispersion scores of reserve and fragment plots were then compared using Welch’s two-sample *t*-tests. As with our analyses of temporal gamma and alpha diversity, we repeated all analyses with the temporal beta diversity of native plants as an added independent variable (i.e., multiple regression models with main effects of treatment and plot-level multivariate dispersion of plant assemblages). Models that included plant temporal beta diversity yielded poorer AIC scores in all cases; thus, we did not include plant temporal beta diversity in our final models.

## Results

In two years of sampling, we collected 12,036 bee specimens belonging to 233 species (190described species and 43 additional morphospecies) in 54 genera and 6 families (S2 Table). Bowl trapping yielded 9,421 specimens (82%) belonging to 168 species (71%), while aerial netting yielded 2,128 specimens (18%) belonging to 179 species (76%). We identified 11,449 specimens (95.1%) to described species, including 485 non-native honey bee workers (*Apis mellifera* L.) and two specimens of non-native wild bees: one female *Megachile rotundata* (Fabricius) and one male *Hylaeus leptocephalus* (Morawitz). A total of 424 specimens (3.5%) was assigned to morphospecies (S2 Table). Species-level identification for this latter set of bees was hindered by the lack of taxonomic revisions and comprehensive reference collections for many bee genera in this region, evidenced by the ongoing discovery of undescribed species (e.g., [49]). Finally, 163 specimens (1.4%) were not identified to species (or morphospecies) as they were rendered unidentifiable beyond genus (or subgenus) due to weathering or other damage, or were male morphospecies that could not be confidently associated with females.

### Temporal gamma diversity

**Fig 2:**
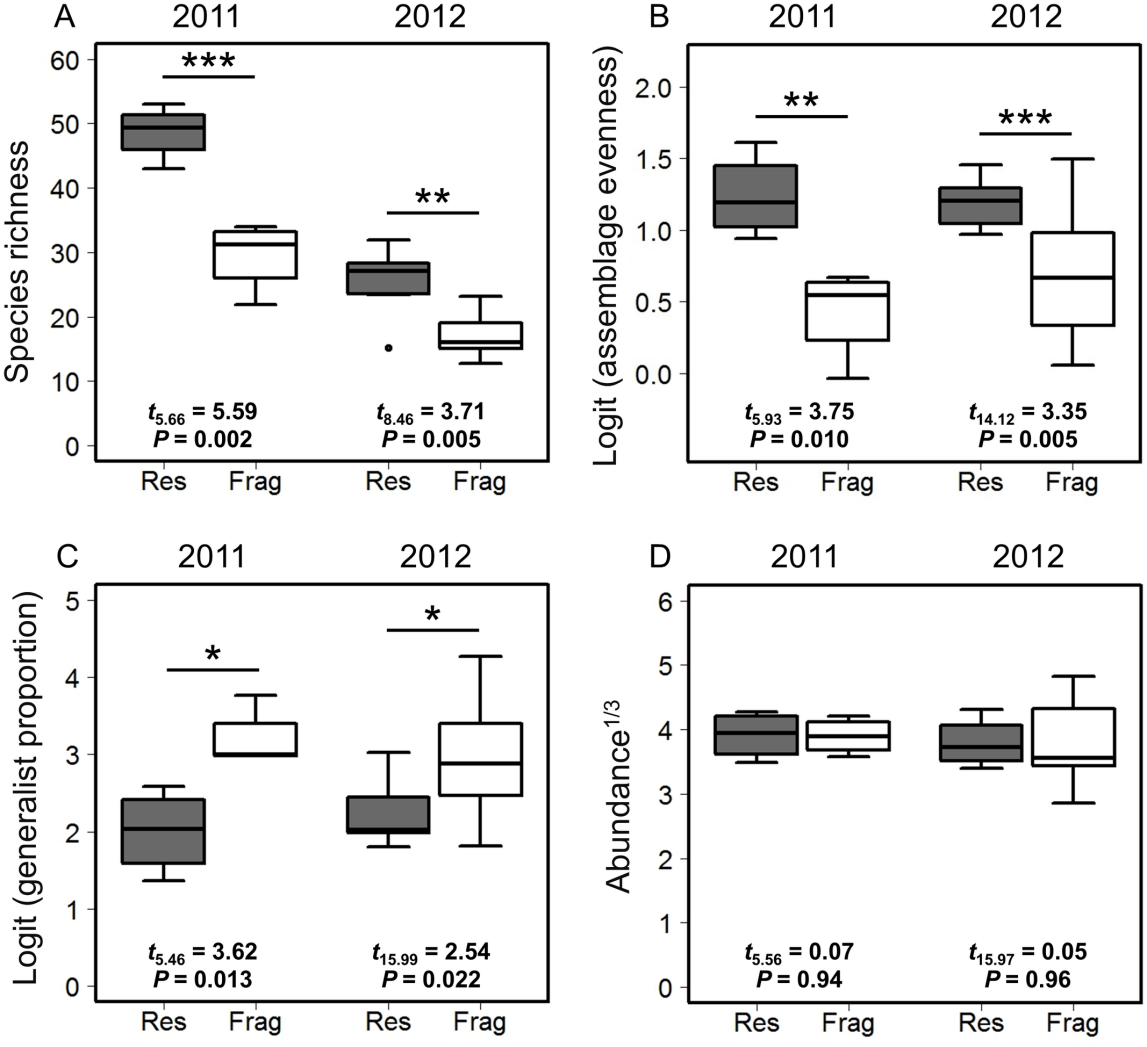
Temporal gamma diversity of native bees in reserve (gray boxes) and fragment plots (white boxes). Box and whisker plots show (A) rarefied species richness, (B) logit-transformed rarefied assemblage evenness (Pielou’s *J*), (C) logit-transformed proportion of individuals belonging to generalist species, and (D) cube root-transformed average number of bees collected per temporal sample. Boxes show central 50% of data and median; whiskers show quantiles ± interquartile range × 1.5, or most extreme values of data, whichever is closest to median; and circles denote outliers. * P < 0.05, ** P < 0.01, *** P < 0.005.

In both years, fragment plots harbored bee assemblages with significantly lower rarefied species richness (Fig 2A; on average 36% lower) as well as lower rarefied assemblage evenness (Fig 2B; on average 18% lower). Fragments also harbored bee assemblages that had higher generalist proportions compared to those in reserves (Fig 2C; on average 7% higher). However, reserves and fragments did not differ in bee abundance in either year (Fig 2D).

### Temporal alpha diversity

**Fig 3:**
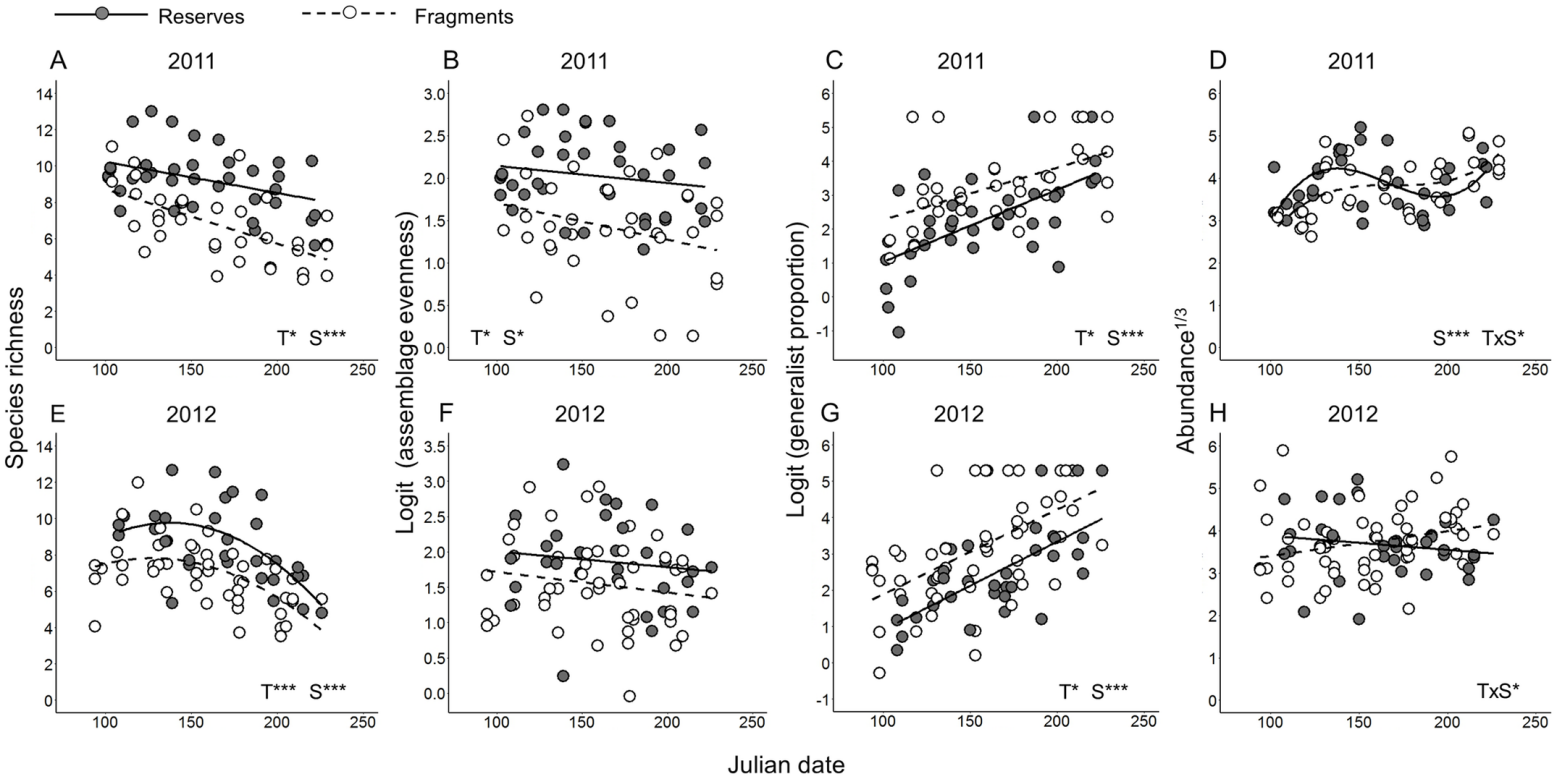
Temporal alpha diversity of native bees in reserve and fragment plots in 2011 (A-D) and 2012 (E-H). Scatterplots show (A,E) rarefied species richness, (B,F) logit-transformed rarefied assemblage evenness (Pielou’s *J*), (C,G) logit-transformed proportions of individuals belonging to generalist species, and (D,H) cube root-transformed number of bees collected per temporal sample. All plots show regression lines to visualize data trends. Significant main effects and interactions from linear mixed-effects models are indicated on each graph: T = treatment, S = temporal sample. * P < 0.05, ** P < 0.01, *** P < 0.005.

Linear mixed-effects models revealed that fragment plots supported bee assemblages with significantly lower rarefied species richness (Fig 3A and 3E; *F*_1,6_ = 12.89, *P* = 0.012 in 2011 and *F*_1,15_ = 13.25, *P* = 0.002 in 2012), with richness decreasing as the Julian date of the sample increased. The relationship between richness and Julian date was linear in 2011 (*F*_1,61_ = 30.77, *P* < 0.0001) and parabolic in 2012 (*F*_1,58_ = 35.99, *P* < 0.0001 for Julian date and *F*_1,58_ = 16.07, *P* = 0.0002 for Julian date^2^). Similarly, assemblage evenness was lower in fragments in 2011 (Fig 3B; *F*_1,6_ = 6.06, *P* = 0.049); the result of this analysis for the 2012 dataset did not attain statistical significance, but nevertheless was consistent with the result of the 2011 dataset (Fig 3F; *F*_1,15_ = 3.97, *P* = 0.065). While assemblage evenness decreased throughout the study period in 2011 (*F*_1,61_ = 5.49, *P* = 0.022), there was no such seasonal effect in 2012 (*F*_1,60_ = 2.09, *P* = 0.15). Generalist proportion was higher in fragments than in reserves in both years (Fig 3C and 3G; *F*_1,6_ = 7.80, *P* = 0.032 in 2011 and *F*_1,16_ = 8.25, *P* = 0.011 in 2012), and increased throughout the study period (*F*_1,62_ = 35.37, *P* < 0.0001 in 2011 and *F*_1,68_ = 64.75, *P* < 0.0001 in 2012).

There was a significant treatment-by-sample interaction for bee abundance in 2011 (Fig 3D; *F*_1,58_ = 5.25, *P* = 0.026 for the interaction involving Julian date; *F*_1,58_ = 0.10, *P* = 0.75 for the interaction involving Julian date^2^; and *F*_1,58_ = 1.48, *P* = 0.22 for the interaction involving Julian date^3^) as well as in 2012 (Fig 3H; *F*_1,68_ = 4.29, *P* = 0.042), wherein bee abundance was generally higher in reserves earlier in the study period and higher in fragments later in the study period. In 2011, abundance varied roughly sinusoidally as the Julian date of the sample increased (*F*_1,58_ = 13.88, *P* = 0.0004 for Julian date; *F*_1,58_ = 0.27, *P* = 0.61 for Julian date^2^; and *F*_1,58_ = 14.30, *P* = 0.0004 for Julian date^3^); however, in 2012, abundance did not vary with temporal sample (*F*_1,68_ = 1.80, *P* = 0.18). In the overall model, bee abundance did not differ between reserves and fragments (*F*_1,6_ = 0.56, *P* = 0.48 in 2011 and *F*_1,16_ = 0.08, *P* = 0.78 in 2012).

### Temporal beta diversity

**Fig 4:**
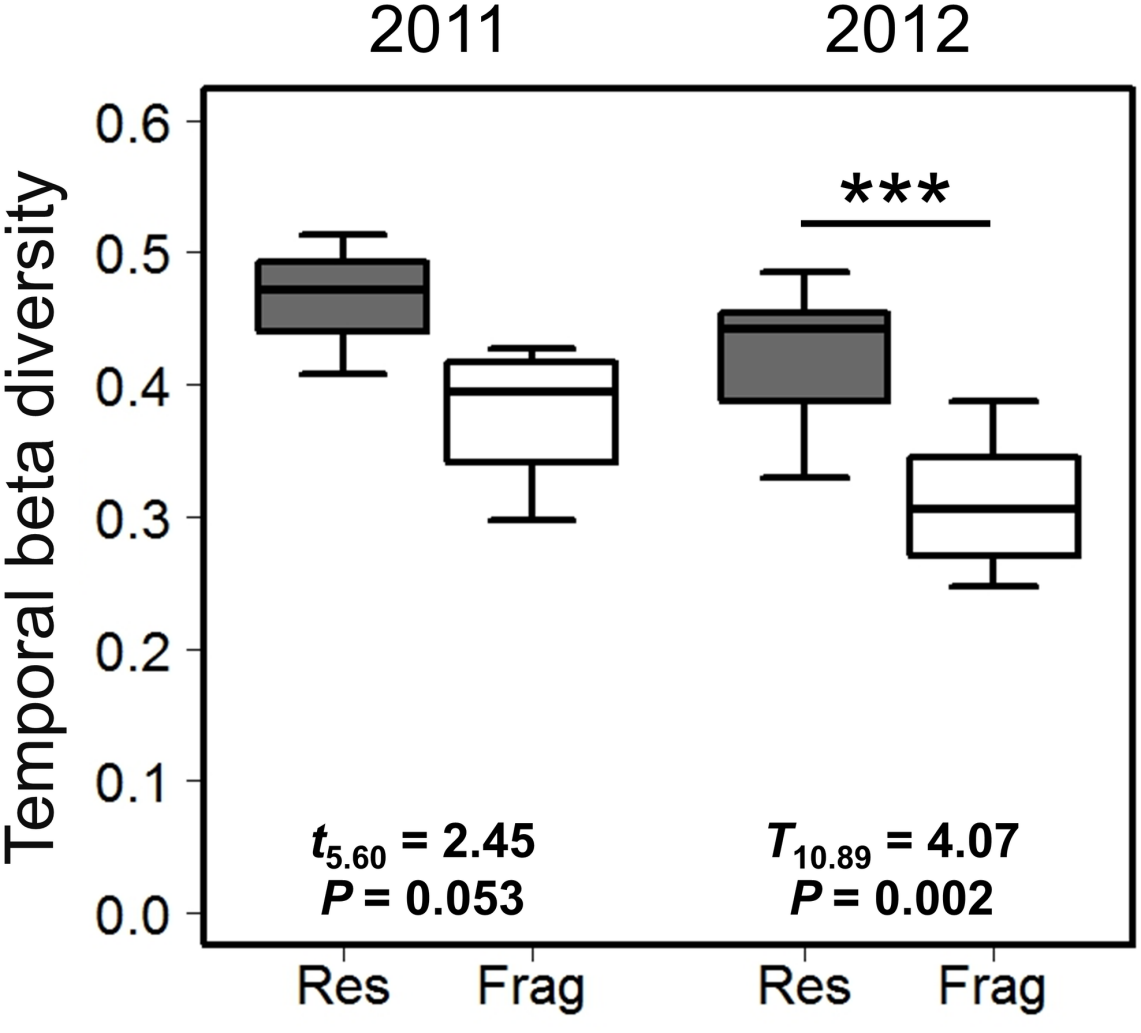
Temporal beta diversity of native bees in reserve (gray boxes) and fragment plots (white boxes). Beta diversity was calculated as the multivariate dispersion of abundance-weighted bee assemblages in distinct temporal samples within each study plot. Boxes, whiskers, and asterisks are as in Fig 2.

The temporal beta diversity of bee assemblages was significantly lower in fragments than in reserves in 2012 (Fig 4; ca. 26% lower). The result of this analysis for the 2011 dataset did not attain statistical significance, but nevertheless was consistent with the result of the 2012 dataset (Fig 4; diversity was ca. 19% lower in fragments compared to reserves).

## Discussion

Across our two years of sampling, we found consistent differences in bee assemblages occurring in reserves and fragments, despite the known tendency for bee faunas to exhibit considerable inter-annual variation at a given locality [47]. Compared to reserves, fragments harbored bee assemblages that were less diverse with respect to all three components of temporal diversity (Figs 2–4). While bee species richness, evenness, abundance, and generalist proportion all varied with time, differences in bee diversity between reserves and fragments were remarkably constant throughout each year’s study period (Fig 3). Individually scrutinizing the three components of temporal diversity allowed for a high-resolution characterization of the temporal structure (Fig 1) of bee assemblages in intact and fragmented habitats; these analyses also yielded further insights into the potential consequences of bee diversity loss for ecosystem function in fragmented habitats in our system.

Reduced species richness is one of the most commonly reported effects of habitat fragmentation on bee assemblages (e.g., [5]). Though our reserve and fragment plots did not differ systematically with respect to the composition of floral resources (S1 Appendix), it is possible that decreased availability of nest sites within foraging distance of key host plants [50,51] or increased vulnerability to demographic stochasticity due to isolation [52] or small population size may have contributed to reduced bee species richness in fragments. Analyses of the temporal gamma and temporal alpha components of bee species richness yielded qualitatively similar results; however, the impact on each of the two temporal diversity components may have distinct implications for the conservation of bees and ecosystem function. The temporal gamma component of bee species richness provides information on the habitat conditions and locations that support the greatest total number of bee species; as such, it is the most useful metric for developing conservation strategies aimed at conserving bee species richness *per se*. On the other hand, the pollination effectiveness of a particular bee species for a particular plant species may depend upon the timing during which the interaction between bees and plants takes place [22] or upon the bee species’ functional complementarity with other, temporally co-occurring pollinator species [23]. Detecting potential impacts of climate change on the phenological matching between bee species and the plants they pollinate [19,53] also requires, at the minimum, examining the composition of bee assemblages at discrete points in time. Thus, as the impacts of climate change on bees intensify [54,55], effective strategies aimed at conserving bees and the ecosystem function they perform should account for both the temporal alpha and gamma components of bee richness.

As with patterns of bee species richness, both temporal gamma and alpha components of bee assemblage evenness suffered declines in fragments relative to reserves. Assemblage evenness is an important driver of ecosystem function (reviewed in [56]), including pollination [57], but remains an under-appreciated aspect of pollinator assemblage dynamics [58]. Reductions in the temporal alpha component of bee assemblage evenness in fragments may result in decreased frequencies of interspecific encounters among bee species; physical encounters between bee species during foraging have been shown to enhance pollination efficiency via altering bee foraging behavior [59]. On the other hand, reductions in the temporal gamma component of bee assemblage evenness may result in a stronger reliance by plant assemblages on a small subset of numerically dominant bee species, and consequently, reduced stability of pollination services [57] (but see [60]).

In contrast to patterns of bee species richness and assemblage evenness, overall bee abundance did not differ between reserves and fragments. This pattern was caused by reserves having higher bee abundance in the spring (April through early June) and fragments having higher bee abundance in the summer (late June through August; Fig 3D and 3H). This treatment-by-sample interaction appears to be driven by the higher relative abundance of generalist bees in fragments (Figs 2C, 3C and 3G); many numerically abundant generalist species in our system (e.g., many primitively eusocial Halictinae species) reach peak abundance between late June and August. Generalist bees may be more tolerant of habitat fragmentation compared to specialists (e.g., [42]) and have been hypothesized to replace the ecosystem function formerly performed by extirpated specialists [61]. However, even though generalists in our study numerically compensated for absent specialists when considering the temporal gamma component of bee abundance (Fig 2D), reduced bee abundance in fragments early in our study period (April through early June) may threaten the pollination of spring-blooming plant species.

Temporal beta diversity represents another under-appreciated metric in ecology [11], and reports on the effects of anthropogenic disturbance on intra-annual turnover of biological assemblages remain rare (e.g., [10,62,63]). In our system, decreased temporal beta diversity in fragments may explain how modest reductions in the temporal alpha component of species richness and assemblage evenness in fragments (Fig 3) translated into more pronounced reductions in the temporal gamma component of each metric (Fig 2). More broadly, decreasing seasonal turnover in an assemblage may result in increasing temporal niche overlap among its constituent species [12], which may in turn decrease the number of distinct temporal niches created by the assemblage. Decreases in the seasonal turnover of bee assemblages may be especially consequential in cases where bee species tend to interact with a set of preferred host plants throughout their activity season even when new plant species begin to bloom as time progresses (e.g., [24]). If temporal host-switching is likewise rare in our system, reduced bee assemblage turnover in fragments may jeopardize the reproduction of certain plant species that occupy specific temporal niches with respect to pollination [23]. Examining the temporal beta diversity of bee assemblages thus appears crucial for understanding mechanisms underlying the impact of anthropogenic disturbance on pollination services.

## Conclusion

Our synthesis of the three components of bee temporal diversity revealed that coastal sage scrub fragments in San Diego County support bee assemblages that (1) have lower species richness and evenness but higher numerical dominance by generalists at any given point in the bee activity season, (2) reach peak abundance later in the bee activity season (late June through August), and (3) exhibit less temporal turnover compared to reserves. Correspondingly, these patterns suggest that plants occurring in scrub fragments may suffer from decreases in (1) the functional complementarity of bee taxa that simultaneously co-occur, (2) floral visitation by bees early in the bee activity season (April through early June), and (3) the number of available temporal niches with respect to pollination. Our research demonstrates the importance of quantifying the gamma alpha, and beta components of temporal diversity when characterizing the impacts of anthropogenic disturbance on seasonal organisms, as well as when predicting how such impacts may influence ecosystem function. As human activity and climate change continue to alter Earth’s ecosystems, it will be increasingly important to document how anthropogenic disturbance impacts assemblage structure and ecosystem functions associated with distinct components of temporal diversity in organisms that exhibit inherent seasonal dynamics.

## Supporting information

S1 Appendix Description and explanation of study sites. (PDF)

S2 Appendix R scripts used in core analyses. (PDF)

S1 Table List of study plots in coastal sage scrub reserves and habitat fragments. (PDF)

S2 Table Species list of bees collected in this study. (PDF)
