# Supplementary material for: Urbanization-induced habitat fragmentation erodes multiple components of temporal diversity in a Southern California native bee assemblage
Source: PLoS One. 2017 Aug 30;12(8):e0184136. doi: 10.1371/journal.pone.0184136 (PMC5576854; doi:10.1371/journal.pone.0184136)
Supplement: S1 Appendix — (PDF) [file pone.0184136.s001.pdf]

# S1 Appendix. Description and explanation of study sites.

**Urbanization-induced habitat fragmentation erodes multiple components of temporal diversity in a Southern California native bee assemblage**

Keng-Lou James Hung, John S. Ascher, and David A. Holway. *PLoS ONE* 2017.

We chose study plots in sites that contained a diversity of native shrubs, dominated by combinations of *Acmispon glaber* (Vogel) Brouillet, *Artemisia californica* Less., *Bahiopsis laciniata* (A. Gray) E. E. Schilling & Panero, *Eriogonum fasciculatum* Bentham, *Malosma laurina* (Nutt.) Abrams, *Rhus integrifolia* (Nutt.) Brewer & S. Watson, and *Salvia mellifera* E. Greene. To the extent possible, we chose sites with minimal invasion by exotic forbs such as *Brassica nigra* (L.) Koch and *Erodium* spp., and exotic grasses such as *Avena* spp. and *Bromus* spp.

Reserve plots were chosen within four distinct reserves: Elliott Chaparral Reserve of the University of California Reserves System (plots ECR1 and ECR2), Mission Trails Regional Park (plots MTE2 and MTI2), the Otay-Sweetwater unit of the San Diego National Wildlife Refuge (plots SWEA and SWI2), and the Tijuana River National Estuarine Research Reserve (plot TRR1). Fragment plots were chosen in the vicinity of reserve plots (maximum distance between fragment and reserve plots < 15 km) and consisted of well-preserved scrub habitat surrounded by urban, residential infrastructure such as roads (two lanes minimum), buildings, and paved lots (and in one instance, a sandy beach). Details on each study plot are given in S1 Table. Permission to perform research was obtained from the University of California Natural Reserves System, the National Wildlife Refuge, the City of San Diego, the City of Chula Vista, and the City of La Mesa. Plots are separated from their nearest neighbors by a minimum of 1.5 km, except for two pairs of plots which are separated from their nearest neighbors by ca. 500 m. Despite the fact that six of our reserve plots represented relatively closely-situated pairs of plots in three natural reserves, we opted to treat them as independent replicates for two reasons. First, Mantel Tests reveal no spatial autocorrelation with respect to bee assemblage composition as calculated by the Bray-Curtis dissimilarity index ($r = 0.073$, $P > 0.05$ in 2011; $r = 0.15$, $P > 0.05$ in 2012). Second, excluding one plot from each pair of closely-situated plots from our analyses did not change our main conclusions.

To verify that our reserve and fragment study plots contained plant assemblages similarly representative of intact coastal sage scrub, we compared reserve and fragment plots with respect to insect-pollinated native plant species richness and the abundance of perennial, insect-pollinated shrubs. We also compared the composition of native plant assemblages in reserve and fragment plots using permutational ANOVAs (PERMANOVAs). Lastly, we also examined the multivariate dispersion of temporal samples of plants as a metric of the temporal turnover of plant assemblages. Our estimates of the presence-absence (in 2011) or number of individuals (2012) of each plant species in bloom did not allow for direct comparisons of the abundance and evenness of floral resources across sites and across plant species because of variation in plant sizes across sites and across species. Thus, we performed PERMANOVAs and calculated multivariate dispersions of plant assemblages based on presence-absence data rather than count data (for calculations of multivariate dispersion, plant species that were blooming during a survey round was scored as “present,” all other plant species were scored as “absent”).

Compared to analyses using abundance-weighted data, analyses using presence-absence data yield results that are lower in resolution but nevertheless qualitatively similar.

Native plant species richness did not differ between reserves and fragments in 2011 (two-sample *t*-test $t_{3.66} = 0.84$, $P > 0.05$) or 2012 ($t_{15.36} = 0.92$, $P > 0.05$). Similarly, shrub abundance did not differ between reserves and fragments in 2012 ($t_{13.97} = 0.67$, $P > 0.05$); shrub abundance was not recorded with sufficient resolution in 2011 to allow comparison between reserves and fragments. Reserves and fragments also did not differ with respect to native plant assemblage composition in 2011 (PERMANOVA $F_{1,6} = 0.88$, $P > 0.05$) or 2012 ($F_{1,16} = 1.25$, $P > 0.05$). Lastly, plant temporal beta diversity did not differ between fragments and reserves in 2011 (two-sample t-*t*est $t_{4.10} = 0.66$, $P > 0.05$) or 2012 ($t_{15.01} = 0.68$, $P > 0.05$).
