# Supplementary material for: Urbanization-induced habitat fragmentation erodes multiple components of temporal diversity in a Southern California native bee assemblage
Source: PLoS One. 2017 Aug 30;12(8):e0184136. doi: 10.1371/journal.pone.0184136 (PMC5576854; doi:10.1371/journal.pone.0184136)
Supplement: S2 Appendix — (PDF) [file pone.0184136.s002.pdf]

S2 Appendix. R scripts used in core analyses.

Urbanization-induced habitat fragmentation erodes multiple components of temporal diversity in a Southern California native bee assemblage

Keng-Lou James Hung, John S. Ascher, and David A. Holway. *PLoS ONE* 2017.

Calculating rarefied Shannon diversity and evenness measures

```
library(vegan)

# Create matrices for storing diversity calculation. 1000 rows for number of iterations, 18
        columns for the 18 sites.
diversmat = matrix(data = 0, nrow=1000, ncol=18)
colnames(diversmat) =  row.names(bees.data.12)
evenmat = matrix(data = 0, nrow=1000, ncol=18)
colnames(evenmat) =  row.names(bees.data.12)

# Loop (repeated for n = reps +1 times) for generating randomly-selected rarefied communities
i <- 0
reps = 999
repeat
{i <- i + 1;

# Generate a single randomly rarefied community and store it in a temporary matrix
a = rrarefy(bees.data.12, 100);

#Calculate Shannon diversity of matrix a and store it in matrix b
b = diversity(a, index="shannon");

#Calculate Pielou’s J evenness of matrix a based on Shannon diversity, and store it in matrix c
c = b/log(specnumber(a));

#Store the single iteration of diversity and evenness into the eventual output matrix
diversmat[i, ] = b;
evenmat[i,] = c;

if (i > reps) break;}

#Calculate the column means of output matrices, which are the results of the Shannon diversity
        and evenness rarefaction iterations.
colMeans(diversmat, na.rm = FALSE, dims = 1)
colMeans(evenmat, na.rm = FALSE, dims = 1)
```

Analyzing temporal alpha diversity using linear mixed-effects models

```
library(nlme)
library(car)        # Used for the logit() function

# First-degree mixed-effects models
model11spp = lme(logit(Evenness) ~ Treatment * Julian, random = ~1|Site, data =alpha.11)
model11spp1 = lme(logit(Evenness) ~ Julian * Treatment, random = ~1|Site, data = alpha.11)

# Extract stats on the fixed effect included second (Julian date) for type II SS
anova(model11spp)

# Extract stats on the fixed effect included second (treatment) for type II SS
anova(model11spp1)


# Orthogonal polynomial mixed-effects models

# Verify that polynomial models have improved AIC scores compared to first-degree models
AIC(lme(Abundance^(1/3) ~ poly(Julian,3) * Treatment, random =  ~1|Site, data = alpha.11,
        method = “ML”))       # 3rd degree orthogonal polynomial model
AIC(lme(Abundance^(1/3) ~ poly(Julian,2) * Treatment, random =  ~1|Site, data = alpha.11,
        method = “ML”))       # 2nd degree orthogonal polynomial model
AIC(lme(Abundance^(1/3) ~ Julian * Treatment, random =  ~1|Site, data = alpha.11, method =
        “ML”))                # 1st degree linear model

# Create 3rd degree orthogonal polynomials from the Julian date independent variable
poly.third.julian.11 = poly(alpha.11$Julian, 3)

# Make data.frame with relevant dependent variable, site name, treatment, and orthogonal
        polynomials of Julian dates
data.poly.11 = cbind(alpha.11$Treatment, alpha.11$Site, alpha.11$Abundance,
        poly.third.julian.11)
colnames(data.poly.11) = c("Treatment", "Site", "Abundance", "Julx1", "Julx2", "Julx3")

# Construct linear models and extract stats from the last-included main effect and interaction
        term for type II SS
anova(lme(Abundance^(1/3)~(Julx1+Julx2+Julx3)*Treatment, random= ~1|Site, data =
        data.poly.11))
anova(lme(Abundance^(1/3)~Treatment*(Julx2+Julx3+Julx1), random= ~1|Site, data =
        data.poly.11))
anova(lme(Abundance^(1/3)~Treatment*(Julx1+Julx3+Julx2), random= ~1|Site, data =
        data.poly.11))
anova(lme(Abundance^(1/3)~Treatment*(Julx1+Julx2+Julx3), random= ~1|Site, data =
        data.poly.11))
```

Calculating multivariate dispersion scores for subsequent comparison via *t*-test

```
library(vegan)

# Construct multivariate dispersion object based on a data.frame with species in columns. Each sample represents one temporal sample from one study site; the first column designates the study site to which the sample belongs.
bees.beta.11 = betadisper(vegdist(bees.alpha.11[,-1], method="bray"), bees.alpha.11$Sites, type="median")

# The average distance to median is the within-site temporal dispersion of points, which is a measure of beta diversity
bees.beta.11
```
