# Supplementary material for: Urbanization-induced habitat fragmentation erodes multiple components of temporal diversity in a Southern California native bee assemblage
Source: PLoS One. 2017 Aug 30;12(8):e0184136. doi: 10.1371/journal.pone.0184136 (PMC5576854; doi:10.1371/journal.pone.0184136)
Supplement: S1 Table — (PDF) [file pone.0184136.s003.pdf]

S1 Table. List of study plots in coastal sage scrub reserves and habitat fragments.

Urbanization-induced habitat fragmentation erodes multiple components of temporal diversity in a Southern California native bee assemblage

Keng-Lou James Hung, John S. Ascher, and David A. Holway. *PLoS ONE* 2017.

Plot	Yr. sampled	Frag / Res	Latitude	Longitude	Internal area (ha)
MTLB1	2011	Fragment	32.800	-117.137	116.69
CFS1	2012	Fragment	32.814	-117.237	36.44
MTS2	2012	Fragment	32.856	-117.188	12.89
MTS3	2012	Fragment	32.787	-117.141	31.96
MTS6	2012	Fragment	32.722	-117.119	52.79
MTS7	2012	Fragment	32.740	-117.086	12.01
SCR	2012	Fragment	32.875	-117.248	36.38
SWS10	2012	Fragment	32.786	-116.989	6.23
TRS1	2012	Fragment	32.632	-117.033	9.19
MTS1A	2011-12	Fragment	32.792	-117.061	2.72
SWS1	2011-12	Fragment	32.750	-117.032	46.55
SWS3	2011-12	Fragment	32.720	-117.078	28.06
ECR1	2012	Reserve	32.892	-117.092	> 500
ECR2	2012	Reserve	32.889	-117.096	> 500
TRR1	2012	Reserve	32.565	-117.126	> 500
MTE2	2011-12	Reserve	32.834	-117.078	> 500
MTI2	2011-12	Reserve	32.842	-117.065	> 500
SWEA	2011-12	Reserve	32.732	-116.956	> 500
SWI2	2011-12	Reserve	32.734	-116.950	> 500
