# Supplementary material for: Urbanization-induced habitat fragmentation erodes multiple components of temporal diversity in a Southern California native bee assemblage
Source: PLoS One. 2017 Aug 30;12(8):e0184136. doi: 10.1371/journal.pone.0184136 (PMC5576854; doi:10.1371/journal.pone.0184136)
Supplement: S2 Table — (PDF) [file pone.0184136.s004.pdf]

S2 Table. List of bee species collected in this study.

Urbanization-induced habitat fragmentation erodes multiple components of temporal diversity in a Southern California native bee assemblage

Keng-Lou James Hung, John S. Ascher, and David A. Holway. *PLoS ONE* 2017.

S2 Table. Species list of bees collected in this study, with their classification as specialists (restricted to pollen on one plant family), generalists, or parasites. Asterisks following species names denote exotic species.

Family	Genus	Species	# in frag.	# in res.	Classification
Andrenidae	*Ancylandrena*	*atoposoma*	0	4	Specialist
	Andrena	*anatolis*	4	0	Specialist
		atypica	0	2	Generalist
		auricoma	9	3	Generalist
		candida	2	0	Generalist
		cerasifolii	1	19	Generalist
		gnaphalii	1	7	Specialist
		oenotherae	0	3	Specialist
		osmioides	0	1	Specialist
		pallidifovea	4	0	Specialist
		parachalybea	1	0	Specialist
		piperi	2	0	Generalist
		prunorum	1	0	Generalist
		sola	1	0	Generalist
		nr. *knuthiana*	1	0	Generalist
	Calliopsis	*mellipes*	0	2	Specialist
		obscurella	0	5	Specialist
		pugionis	4	31	Specialist
		rhodophila	44	91	Generalist
	Macrotera	*tristella*	95	65	Generalist
	Panurginus	sp. 1	0	2	Generalist
	Perdita	*californica*	0	10	Specialist
		claypolei australior	21	43	Specialist
		eriastri	8	0	Specialist
		fieldi	0	5	Specialist
		hirticeps hirticeps	1	0	Specialist
		interrupta	0	14	Specialist
		minima	2	0	Specialist
		rhois rhois	105	112	Generalist
		trisignata	0	7	Specialist

Family	Genus	Species	# in frag.	# in res.	Classification
Apidae	*Anthophora*	*crotchii*	0	1	Generalist
		curta	22	9	Generalist
		flavocincta	0	6	Generalist
		urbana	4	5	Generalist
	Anthophorula	*albicans*	0	5	Generalist
		nitens	87	143	Generalist
		torticornis	242	253	Generalist
	Apis	*mellifera**	325	160	Generalist
	Bombus	*californicus*	8	34	Generalist
		crotchii	0	8	Generalist
		melanopygus	1	9	Generalist
		vosnesenskii	16	26	Generalist
	Brachynomada	*annectens*	11	15	Parasite
	Ceratina	*acantha*	156	58	Generalist
		arizonensis	498	212	Generalist
		nanula	15	35	Generalist
		punctigena	4	0	Generalist
	Diadasia	*australis californica*	45	8	Specialist
		bituberculata	32	50	Specialist
		diminuta	0	2	Specialist
		laticauda	2	41	Specialist
		martialis	1	2	Specialist
		nitidifrons	23	45	Specialist
		ochracea	47	49	Specialist
		opuntiae	22	1	Specialist
		rinconis rinconis	11	18	Specialist
	Epeolus	*compactus*	1	2	Parasite
	Eucera	*dorsata*	0	13	Generalist
		edwardsii	0	1	Generalist
		tricinctella	1	361	Generalist
		virgata	0	1	Generalist
	Habropoda	*depressa*	3	0	Generalist
		miserabilis	0	1	Generalist
		tristissima	0	14	Generalist
	Holcopasites	*ruthae*	0	1	Parasite
	Leiopodus	*singularis*	0	15	Parasite
	Melecta	*edwardsii*	0	1	Parasite
	Melissodes	*communis alopex*	11	63	Generalist
		lupina	35	33	Specialist
		montana	13	8	Specialist
		paroselae	0	3	Generalist
		personatella	0	6	Specialist
		plumosa	5	25	Specialist

Family	Genus	Species	# in frag.	# in res.	Classification
Apidae	*Melissodes*	*stearnsi*	0	4	Specialist
		tepida	0	1	Generalist
		tessellata	5	17	Generalist
		tribas	0	3	Specialist
		velutina	1	11	Generalist
		sp. nov. 1	0	6	Specialist
	Neopasites	sp. 1	0	6	Parasite
		sp. 2	0	3	Parasite
	Nomada	sp. 1	1	0	Parasite
		sp. 2	0	1	Parasite
		sp. 3	0	3	Parasite
		sp. 4	9	5	Parasite
	Peponapis	*pruinosa*	3	1	Specialist
	Tetraloniella	*davidsoni*	9	9	Specialist
		pomonae	38	2	Specialist
		sp. nov. 1	0	1	Specialist
	Triepeolus	*californicus*	3	4	Parasite
		matildae	0	5	Parasite
		melanarius	4	7	Parasite
		utahensis	5	2	Parasite
		simplex grp. sp. 1	1	0	Parasite
	Triopasites	*penniger*	0	4	Parasite
	Xeromelecta	*californica*	0	1	Parasite
	Xylocopa	*varipuncta*	2	1	Generalist
Colletidae	*Colletes*	*intermixtus*	1	0	Generalist
		louisae	0	1	Generalist
		slevini	5	17	Generalist
		wootoni	15	9	Generalist
		aff. *deserticola*	10	11	Specialist
	Hylaeus	*episcopalis metzi*	4	8	Generalist
		*Leptocephalus**	0	1	Generalist
		mesillae cressoni	40	17	Generalist
		polifolii	46	19	Generalist
		rudbeckiae	4	1	Generalist
		verticalis	0	1	Generalist
Halictidae	*Agapostemon*	*femoratus*	0	19	Generalist
		melliventris	0	1	Generalist
		texanus	926	499	Generalist
	Augochlorella	*pomoniella*	10	151	Generalist
	Conanthalictus	*bakeri*	0	3	Specialist
	Dieunomia	*nevadensis angelesia*	0	4	Generalist
	Dufourea	*australis*	23	20	Specialist
		brevicornis	0	2	Specialist

Family	Genus	Species	# in frag.	# in res.	Classification
Halictidae	*Dufourea*	*mulleri*	0	1	Specialist
		rhamni	9	31	Specialist
		scintilla	8	0	Specialist
		aff. *sandhouseae*	0	25	Specialist
		cf. *saundersi*	0	1	Specialist
	Halictus	*farinosus*	101	161	Generalist
		ligatus	30	105	Generalist
		rubicundus	0	1	Generalist
		tripartitus	1649	639	Generalist
	Lasioglossum	*albohirtum*	2	0	Generalist
		argemonis	3	17	Generalist
		brunneiventre	241	11	Generalist
		imbrex	26	26	Generalist
		incompletum	643	359	Generalist
		knereri	5	4	Generalist
		mellipes	1	1	Generalist
		microlepoides	83	56	Generalist
		nevadense	58	43	Generalist
		nigrescens	94	42	Generalist
		ovaliceps	2	4	Generalist
		pacificum	1	2	Generalist
		petrellum	6	1	Generalist
		punctatoventre	33	41	Generalist
		sisymbrii	53	55	Generalist
		titusi	0	9	Generalist
		aff. *nevadense*	25	16	Generalist
		cf. *macroprosopum*	60	9	Generalist
		cf. *perparvum*	4	0	Generalist
		cf. *robustum*	4	45	Generalist
		(*Dialictus*) sp. 1	103	0	Generalist
		(*Dialictus*) sp. 2	1	0	Generalist
		(*Dialictus*) sp. 3	1	0	Generalist
		(*Dialictus*) sp. 4	0	3	Generalist
		(*Dialictus*) sp. 5	0	3	Generalist
		(*Evylaeus* s. l.) sp. 1	1	0	Generalist
		(*Evylaeus* s. l.) sp. 2	1	0	Generalist
		(*Evylaeus* s. l.) sp. 3	0	5	Generalist
	Micralictoides	*altadenae*	1	0	Specialist
		chaenactidis	8	0	Specialist
		ruficaudus	2	46	Specialist
	Sphecodes	*arvensiformis*	7	4	Parasite
		sp. 1	3	9	Parasite
		sp. 2	9	4	Parasite

**Family**	**Genus**	**Species**	**# in frag.**	**# in res.**	**Classification**
Halictidae	*Sphecodes*	sp. 3	0	2	Parasite
		sp. 4	4	3	Parasite
		sp. 5	2	2	Parasite
		sp. 6	0	1	Parasite
		sp. 7	0	1	Parasite
		sp. 8	1	0	Parasite
		sp. 9	1	0	Parasite
		sp. 10	1	0	Parasite
		sp. 11	1	0	Parasite
Megachilidae	*Anthidiellum*	*notatum robertsoni*	1	0	Generalist
	Anthidium	*collectum*	2	16	Generalist
		edwardsii	0	5	Generalist
		illustre	0	4	Generalist
		jocosum	1	112	Generalist
		mormonum	0	1	Generalist
		palliventre	0	9	Generalist
		utahense	0	1	Generalist
	Ashmeadiella	*bucconis denticulata*	1	10	Specialist
		californica californica	10	53	Generalist
		cubiceps	0	2	Generalist
		foveata	4	10	Generalist
		meliloti meliloti	2	1	Generalist
		rufipes	0	12	Generalist
		rufitarsis	1	2	Specialist
		salviae	3	10	Generalist
		titusi	0	5	Specialist
	Atoposmia	*copelandica arefacta*	0	1	Specialist
		hemizoniae	1	1	Specialist
	Chelostoma	*californicum*	0	1	Specialist
		phaceliae	0	10	Specialist
	Coelioxys	sp. 1	0	2	Parasite
		sp. 2	0	1	Parasite
		sp. 3	0	1	Parasite
	Dianthidium	*dubium dilectum*	1	22	Generalist
		pudicum consimile	10	43	Generalist
	Dioxys	*productus cismontanicus*	0	10	Parasite
	Heriades	*occidentalis*	4	2	Generalist
	Hoplitis	*albifrons maura*	0	7	Generalist
		cryptanthae	0	1	Specialist
		fulgida platyura	1	0	Generalist
		grinnelli	1	31	Generalist
		howardi	0	2	Specialist
		hypocrita	1	0	Generalist

**Family**	**Genus**	**Species**	**# in frag.**	**# in res.**	**Classification**
Megachilidae	*Hoplitis*	*remotula*	0	2	Specialist
		seminigra	0	2	Specialist
		semirubra	1	5	Specialist
	Megachile	*coquilletti*	2	8	Generalist
		fidelis	9	19	Generalist
		frugalis pseudofrugalis	2	1	Generalist
		lippiae	0	2	Generalist
		montivaga	2	4	Generalist
		onobrychidis	0	7	Generalist
		parallela	1	11	Specialist
		*rotundata**	1	0	Generalist
		subnigra angelica	4	1	Specialist
		cf. *seducta*	0	7	Specialist
	Osmia	*albolateralis*	0	1	Generalist
		californica	0	1	Specialist
		clarescens	15	17	Generalist
		coloradensis	22	7	Specialist
		gabrielis	1	4	Generalist
		granulosa	1	13	Generalist
		grinnelli	0	1	Specialist
		kincaidii	0	2	Generalist
		mixta	1	0	Generalist
		montana quadriceps	0	4	Specialist
		nemoris	2	4	Generalist
		sp. 1	0	1	Generalist
	Protosmia	*rubifloris*	1	0	Generalist
	Stelis	*micheneri*	0	1	Parasite
		montana	0	4	Parasite
		trichopyga	0	1	Parasite
		cf. *hurdi*	0	1	Parasite
Melittidae	*Hesperapis*	*ilicifoliae*	0	6	Specialist
